# A Multidisciplinary Quality Improvement Initiative to Improve Hospital Throughput in a Pediatric Tertiary Care Center

**DOI:** 10.1097/pq9.0000000000000857

**Published:** 2025-12-01

**Authors:** Satvika Mikkilineni, Rebecca Cantu, David M. Spiro, Shelly Keller, Justin Criddle, Ryan Kwong, Sara C. Sanders

**Affiliations:** From the * Section of Pediatric Hospital Medicine, Arkansas Children’s Hospital, Little Rock, Ark.; † Department of Pediatrics, University of Arkansas for Medical Sciences, Little Rock, Ark.; ‡ Section of Pediatric Emergency Medicine, Arkansas Children's Hospital, Little Rock, Ark.; § Department of Quality and Innovation, Nemours Children’s Health, Orlando, Fla

## Abstract

**Introduction:**

Overcrowding in the emergency department (ED) can lead to patient delays, increased medical errors, and increased left without being seen (LWBS) rates. Identifying systemic factors that hinder patient throughput can be beneficial to optimizing patient flow and thereby reducing LWBS rates. This quality improvement project aimed to improve hospital throughput in our pediatric tertiary care center through a multidisciplinary systemic approach.

**Methods:**

This study included patients admitted from the ED to an inpatient medical-surgical unit on the pediatric hospital medicine service. This project was a multidisciplinary effort with participation from the ED, hospital medicine, operations center, hospital administration, and nursing, who met biweekly to review data, assess the impact of changes, and discuss new interventions. The project’s key drivers were the duration of the admission process, a shared mental model of admission and discharge processes, and inpatient efficiency. The team conducted a total of 10 interventions during the study period.

**Results:**

The percentage of patients admitted within 90 minutes increased from the baseline of 18% to 42% by the end of the study period. The escalation of care and LWBS rates decreased by the end of the study.

**Conclusions:**

A multidisciplinary approach to hospital throughput, incorporating initiatives from the ED and inpatient units for admission and discharge processes, along with the support from ancillary services, can significantly enhance patient flow throughout the hospital system.

## INTRODUCTION

Patients present to emergency departments (EDs) for acute medical management, requiring medical personnel and ancillary staff to work collaboratively to manage problems.^1^ Patients requiring admission from the ED encounter bottlenecks during the transition of care due to overcrowded hospital systems, causing delays in patient flow and strain on the ED. This overcrowding leads to medical errors, financial loss, and higher left without being seen (LWBS) rates.^2–5^ In 2020, our institution’s opportunity cost for patients who left without treatment for more than 2 months was $494,500–$714,350.

Traditionally, improving patient throughput has been addressed by improving flow in the ED without analyzing patient flow throughout the rest of the hospitalization. One tertiary care center with a level 1 pediatric trauma center renovated its waiting room and created a front-end team of providers who could more readily evaluate patients, leading to a 22-minute improvement in the mean time to first physician evaluation for Emergency Severity Index 3 patients.^6^ Long wait times and an increasing number of visits to the ED have motivated hospitals to increase throughput from the ED to the inpatient floor. Recently, Lean principles have been implemented to address process barriers and improve patient flow systemically.^7^ Previous throughput work focused on improving isolated processes. It did not consider the interconnected nature of the entire hospital system.^8^ Identifying systemic barriers can be beneficial to optimizing patient flow and reducing the LWBS rate.

One prior quality improvement (QI) project aimed at improving the time from admission decision to admission was nursing-focused and based on admissions to a short-stay hospital unit.^9^ The institution cited a lack of a standard admission process, absence of visual indicators of patients ready for admission, and fragmented communication between teams as the main causes of inefficient throughput. Once the admission process addressed these barriers, the admission time decreased by 24 minutes.^9^ Another study highlighted the impact of increasing nonpeak discharges (occurring between 5 pm and 11 am).^10^

Our QI project aimed to improve hospital throughput in our pediatric tertiary care center through a multidisciplinary approach that involved understanding patient flow and systematically identifying bottlenecks, using the Theory of Constraints.^11^ The Specific, Measureable, Achievable, Relevant, and Time-bound aim was to increase the percentage of patients admitted from the ED to the medical-surgical (Med-Surg) units within 90 minutes from admission decision to admission, from a baseline of 18% in January 2022 to 40% by October 2023. We tracked the LWBS rate as a secondary measure, and the escalation of care (EOC) served as the balancing measure.

## METHODS

### Patient Population

This initiative took place in a 336-bed, free-standing children’s hospital located in a rural southern state. The hospital is part of the only pediatric hospital system in the state, featuring a level 1 trauma center, a pediatric intensive care unit (PICU), a cardiovascular intensive care unit, and a level 4 neonatal intensive care unit. The hospital manages an operations center (OC), a centralized transfer hub for consultations and patient transfers into the system. The hospital averages 67,000 ED patient visits and 16,000 inpatient admissions annually, with 60%–70% of Med-Surg admissions assigned to hospital medicine (HM). Patients were included if admitted from the ED to a Med-Surg unit on the pediatric HM service. Patients were excluded if they were admitted to the PICU, cardiovascular intensive care unit, or neonatal intensive care unit, went directly to the operating room, or were admitted directly from another facility.

### Planning the Interventions

**Fig. 1.**
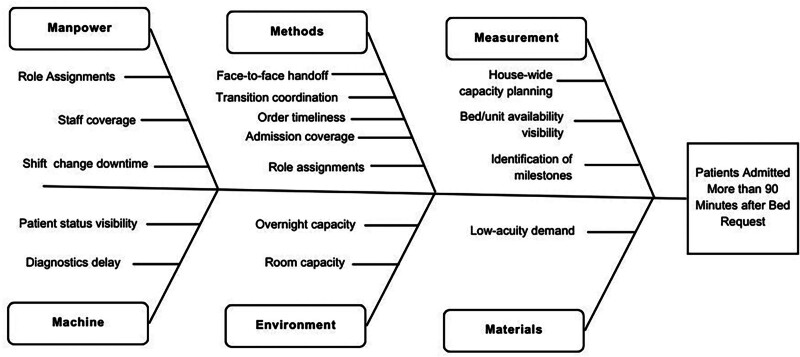
Fishbone diagram detailing bottlenecks contributing to poor hospital throughput.

**Fig. 2.**
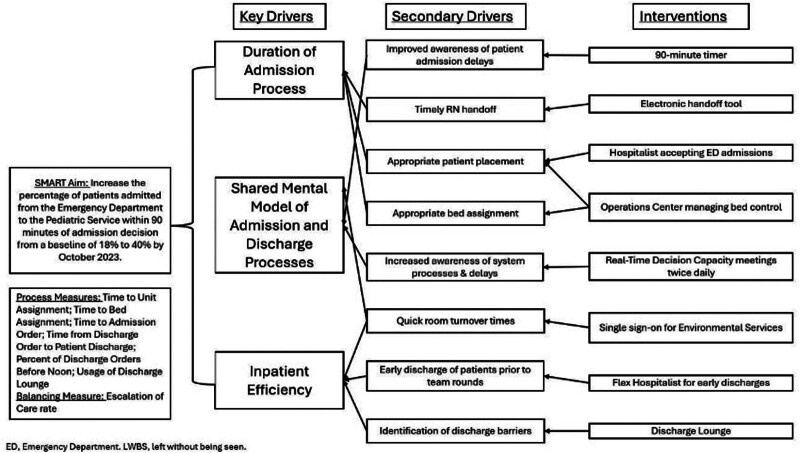
Key drivers diagram of hospital throughput. RN, registered nurse.

Individuals from multiple disciplines in the hospital formed the Throughput Steering Committee with a charter to improve patient throughput from ED presentation through inpatient discharge. Process improvement engineers and senior leadership (including the chief medical officer, chief nursing officer, patient transport, case management, nursing leadership, ED physicians, and HM physicians), all participated. This group identified the root causes of bottlenecks in the throughput process by developing a fishbone diagram (Fig. 1) and a key drivers diagram (Fig. 2), and chose the percentage of patients arriving at their unit within 90 minutes or less from the admission decision as the primary outcome measure. Senior hospital leadership sponsored the committee with hospital-wide goals based on throughput work. The Throughput Committee met twice a month to review data, assess the impact of changes, and discuss new interventions, utilizing the A3 process approach for mapping, implementing, and owning various interventions. The time from admission decision to admission was chosen as a primary measure of throughput because successful improvement in this metric requires input from processes upstream and downstream (ie, ED flow, inpatient workflows, and efficient discharging). This measure aligned the efforts of the admitting teams, support services, inpatient nursing, and the ED team. The committee set 90 minutes as the goal for time to admission, compared with our preintervention baseline of 149 minutes. The project’s key drivers were the duration of the admission process, a shared mental model of admission/discharge processes, and inpatient efficiency.

### Interventions

#### Duration of Admission Process

Secondary drivers contributing to an efficient admission process include timely nursing handoff from the ED to the inpatient floor with appropriate patient placement and bed assignment. To facilitate a timely handoff between ED and Med-Surg nurses, the nursing team implemented electronic nursing handoffs using the secure chat feature in the electronic health record (EHR), Epic (Epic Systems, Verona, WI). This method was implemented if multiple attempts at verbal handoff failed. It enabled asynchronous communication between ED staff and floor nurses. Ultimately, we discontinued this intervention due to poor acceptance from the inpatient nursing teams.

Incorrect patient placement and mismatches in bed placement can contribute to long admission times due to uneven patient distribution among units. To address this process, the OC nurses began assigning both units and beds based on preselected preferences determined by the unit and noted in the EHR. Previously, the Admissions Department, a nonclinical entity, made unit assignments, and nursing unit leaders were responsible for assigning beds. The OC nurses are clinically experienced and focus on both internal and external patient flow. Throughout this process, they became more integrated into internal patient movement.

Long room turnovers contribute to prolonged admission times. The environmental services (EVS) staff previously logged into workstations to identify rooms ready for turnover. To improve efficiency, a single sign-on system was implemented in the electronic interface, allowing EVS to more easily identify rooms requiring cleaning.

#### Shared Mental Model of Admission/Discharge Processes

During Steering Committee discussions, multidisciplinary members identified barriers, including a lack of a shared systemic understanding of the throughput process, such as bed availability, steps in the admission/discharge processes, and delays throughout the system. Although residents rotated in both the ED and inpatient units, most other providers were not aware of workflows in other units. For example, common reasons for delayed admission from the ED to the floor included a lack of floor nurse availability to receive handoffs and the unavailability of the admitting team due to rounds/patient care. To increase system-wide awareness of patient throughput in the hospital, twice-daily multidisciplinary huddles, known as real-time demand/capacity meetings, were implemented to discuss throughput barriers and provide on-demand solutions with broad departmental representation. (**See figure, Supplemental Digital Content 1**, which displays details about RTDC meetings, https://links.lww.com/PQ9/A721.)

At the beginning of the institution’s throughput project, the committee’s focus was on improving ED workflow, as historically, that had been the approach to improve hospital throughput. Long wait times and patient bottlenecks throughout the ED and waiting room raised concerns that admissions were being delayed, increasing patient safety risks. Therefore, a secondary driver contributing to ED efficiency is the quick evaluation and determination of disposition for low-acuity patients. The creation of the clinical decision unit, vertical ED, and provider-in-triage helped evaluate patients who could have a disposition determined quickly without prolonging the time for evaluation in the ED. These initiatives could also aid in evaluating large volumes of patients while simultaneously preventing an increase in the LWBS rate. We also used the EHR to display a 90-minute timer on the ED dashboard, increasing awareness of patients waiting to be admitted.

#### Inpatient Efficiency

Secondary drivers of inpatient efficiency include rapid room turnover, early identification of discharge barriers, and timely patient discharge. The introduction of single sign-on for EVS staff addressed the prolonged turnover times, allowing patients to transfer efficiently from the ED to the Med-Surg unit.

The time required for the admitting service to place admission orders for patients coming to the Med-Surg unit is a previously identified barrier to patient throughput at our institution. The cultural practice was that the ED placed a bed request order, triggering a page to the admitting service, who saw the patient in the ED and wrote admission orders after discussion with the supervising hospitalist attending. The care of other patients often caused delays in this process, leading to increased ED boarding times and downstream effects such as long waiting room times. Patient conditions sometimes worsened from the admission request to the primary team assessment and from the patient’s arrival on the floor, resulting in urgent reassessments and EOC requests. To address these issues, a hospitalist staffed in the OC discussed requested admissions with the ED physicians and then placed admission orders promptly to facilitate movement from the ED. The admitting hospitalist team saw the patient upon arrival on the inpatient floor. If the OC hospitalist had concerns that a patient needed a higher level of care/intervention before admission, the admission was paused and reassessed. This process was initially in place 8 hours per day, but given the trial’s success, it expanded to 16 hours per day 7 months after initiation.

Patients who met discharge criteria in the morning before team rounds typically waited for the rounds to occur before being discharged by their primary team. The hospitalist teams at our institution are not geographically assigned; therefore, the 3 teaching teams and the hospitalist-only team round on multiple units. They often begin rounds with the sickest and newest patients, resulting in longer waits for patients who are ready for discharge. We created the “Flex Hospitalist” role to prioritize discharging patients who were ready to go before or during team rounds so the room could be quickly turned over for new admissions. Secondary duties for this role included seeing direct admissions during team rounds, assessing potential ED admissions that were paused by the OC hospitalist, educational roles, and general pediatric consults.

Finally, patients who were discharged but awaiting a meal, outpatient medication, or transportation could leave their inpatient rooms and stay in the discharge lounge. The discharge lounge was a multiuse area within the hospital where patients who were medically cleared for discharge could go to await the resolution of any discharge barriers. By creating a physical space for these patients to wait outside of their hospital room, EVS and nursing could more quickly prepare for new admissions.

### Measures

Data were collected from January 2022 to October 2023, spanning 10 Plan-Do-Study-Act (PDSA) cycles, with baseline data gathered 6 months before the study period (Table 1). The outcome measure was the percentage of patients admitted from the ED to the Med-Surg units on the HM service within 90 minutes of the bed request order. Process measures included the time, in minutes, from the bed request in the ED to patient arrival on the inpatient unit. For this project, these measures were broken down into time in minutes, from unit assignment to bed assignment, and to admission order, which were analyzed using X-bar S charts. Additional throughput process measures included the time in minutes from discharge order entry to discharge, the percentage of patients with discharge orders placed before noon, the number of patients using the discharge lounge, and the LWBS rate. The balancing measure was defined as EOC within 6 hours of admission to a Med-Surg unit, which was considered a patient transfer from a Med-Surg unit to the PICU or intermediate care unit due to a change in clinical status.

**Table 1.** PDSA Cycles with Interventions and Involved Departments

PDSA Cycle No.	Date(s)	Initiative Name	Description	Involved Departments
1	May 2022	Single sign-on for EVS	Single sign-on to the electronic interface for the EVS team to have easier access to identifying rooms that require cleaning	ED, HM, OC, A
2	August 2022–December 2022	Electronic nursing handoff	Electronic nursing handoff in the EHR for admitted patients from the ED nurse to the Med-Surg floor nurse	N
3	August 2022	90-min timer on ED admit track board	A timer was added to the ED admission track board in the EHR that started with the admission request and would turn red when the 90-min time goal to admission was exceeded	ED, HM, OC, N, A
4	October 2022	Clinical unit assignments	OC nurses began assigning inpatient units for all Med-Surg admissions and transfers	OC, N
5	November 2022	Hospitalist acceptance of ED admissions	ED admissions to the hospitalist service were accepted through an attending-to-attending brief conversation with admission order placement that facilitated movement from the ED; initially, 8 h/d	ED, HM, OC, N
6	December 2022	Flex Hospitalist	A Flex Hospitalist discharges patients ready for discharge early in the morning, reducing reliance on rounding teams to complete this task during rounds	HM
7	June 2023	Expansion hours of hospitalist acceptance of ED admissions	Expanded PDSA cycle Nos. 7–16 h/d	ED, HM, OC, N
8	September 2023	Real-time demand capacity twice-daily huddles	Discuss throughput barriers in real time and provide on-demand solutions; these huddles include broad department representation	ED, HM, OC, N, A
9	September 2023	Discharge lounge	Discharge lounge space was developed to allow a space for noninfectious patients who are discharged, but awaiting a meal, medication, or transportation to wait	ED, HM, OC, N, A
10	October 2023	Clinical bed assignments	OC nurses began assigning specific bed spaces to all Med-Surg admissions and transfers using preferences created by the floor team and noted in the EHR	OC, N

A, ancillary staff; N, nursing.

### Study of Interventions

We used the Model for Improvement to design the study. Data measurements gathered throughout the PDSA cycles guided decisions on future cycle iterations. The Throughput Steering Committee met twice monthly to review data, discuss PDSA cycles, and disseminate changes throughout the hospital. After evaluating each cycle, the Steering Committee decided to continue, stop, or change the PDSA cycle intervention. The data were plotted on statistical process control charts, also known as run charts, and analyzed using the rules for identifying special cause variation. A centerline shift occurred after 8 consecutive points on 1 side of the centerline for control charts and after 7 consecutive points on run charts.^12,13^ Data monitoring continued after interventions to ensure the sustainability of results. We created figures and tables using Microsoft Excel for Microsoft 365 (Version 2501).

## RESULTS

**Fig. 3.**
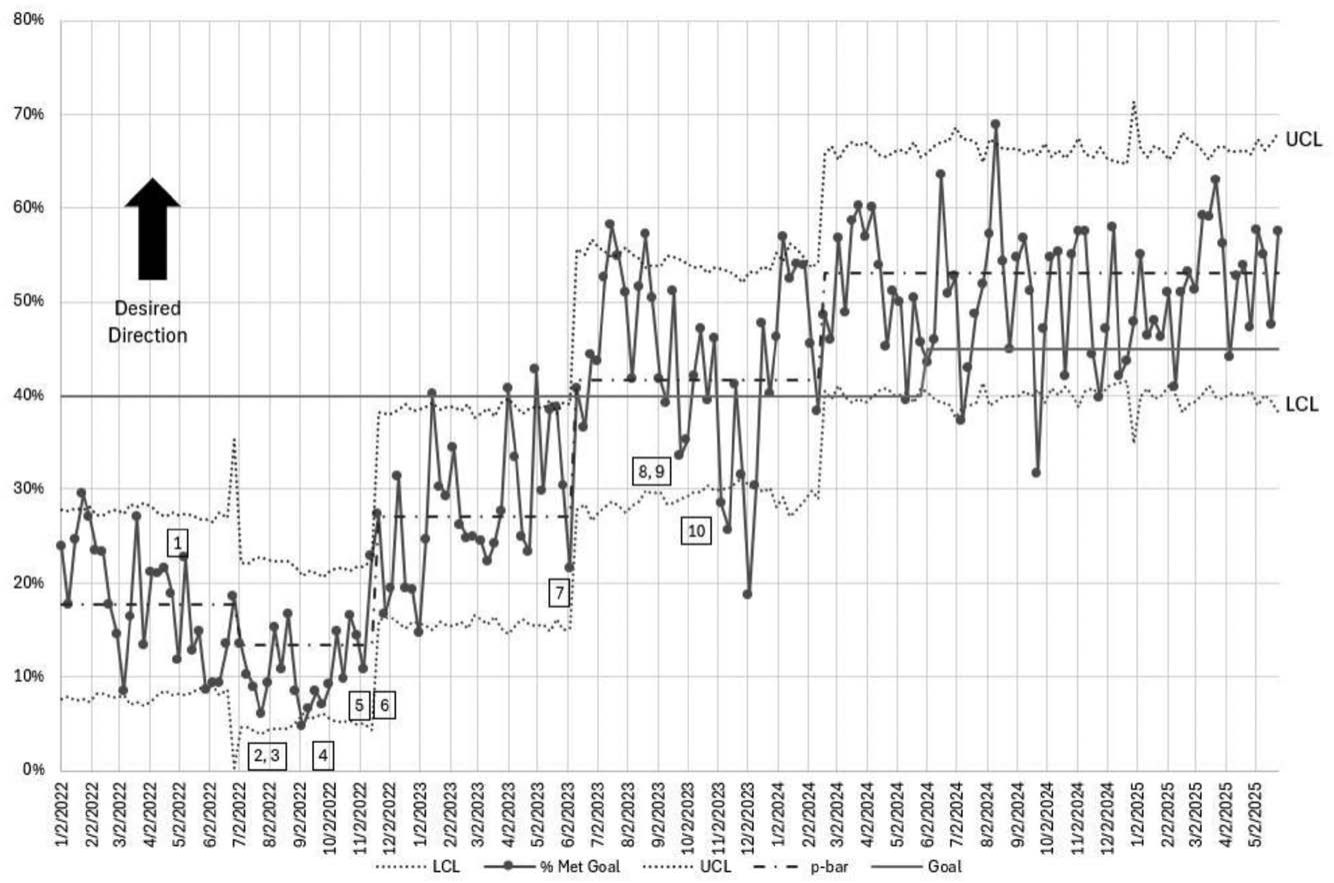
P chart of percentage of admission within 90 minutes of bed request for patients admitted to Med-Surg unit on the pediatric service. Intervention key: 1, single sign-on for EVS; 2, electronic nursing handoff; 3, 90-minute timer on ED track board; 4, clinical unit assignments; 5, hospitalist acceptance of ED admissions; 6, Flex Hospitalist; 7, expansion hours of hospitalist acceptance of ED admissions; 8, real-time demand capacity twice-daily huddles; 9, discharge lounge opening; 10, clinical bed assignments. LCL, lower control limit; UCL, upper control limit.

The percentage of patients admitted within 90 minutes increased from the baseline of 18% to 42% by the end of the study period (Fig. 3). Bed request to admission order time improved from 76 to 43 minutes. Bed request to bed assignment time improved from 75 to 66 minutes.

**Fig. 4.**
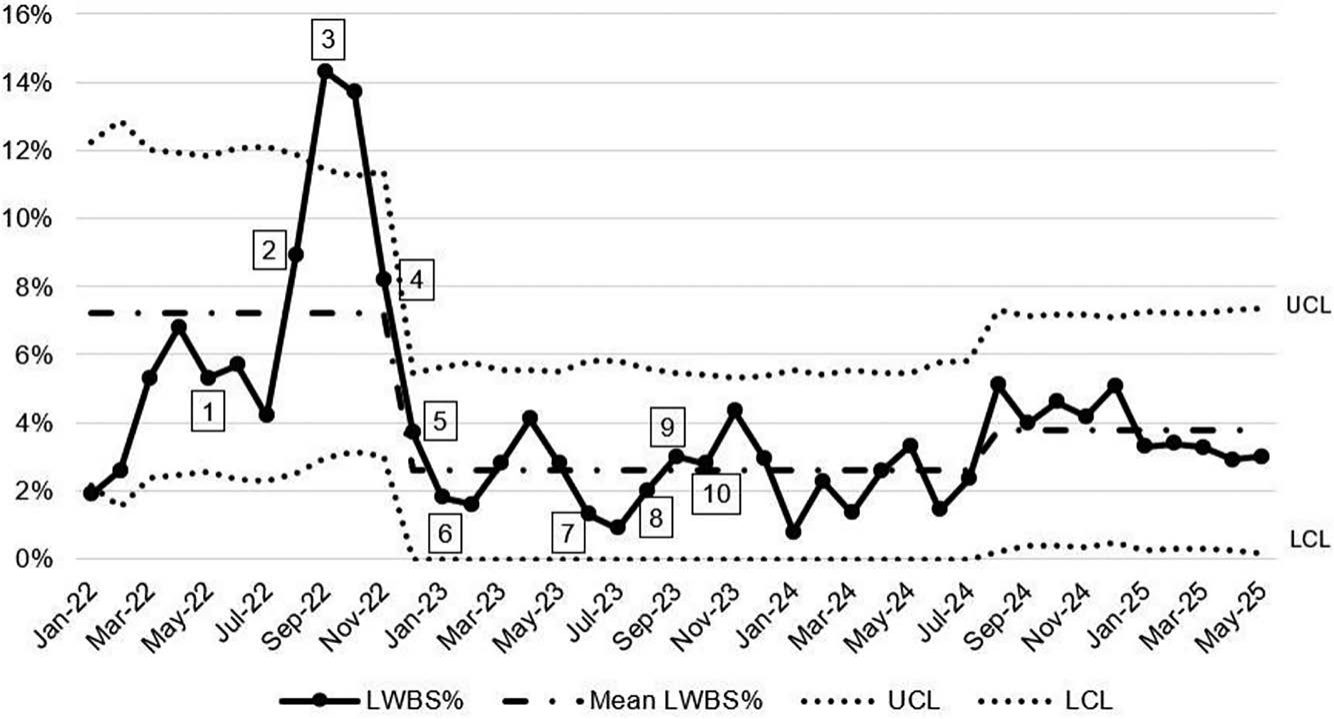
Percentage of patients LWBS from the ED. Intervention key: 1, single sign-on for EVS; 2, electronic nursing handoff; 3, 90-minute timer on ED track board; 4, clinical unit assignments; 5, hospitalist acceptance of ED admissions; 6, Flex Hospitalist; 7, expansion hours of hospitalist acceptance of ED admissions; 8, real-time demand capacity twice-daily huddles; 9, discharge lounge opening; 10, clinical bed assignments. LCL, lower control limit; UCL, upper control limit.

The mean time from discharge order entry to discharge improved from 113 to 97 minutes. The percentage of pediatric service patients with discharge orders before noon averaged 50% after the discharge efficiency interventions. The number of patients using the discharge lounge was 30 (approximately 1.67 per wk) over the first 8 months of implementation. The mean LWBS rate from the ED decreased throughout the study from 4.8% to 2.4% (Fig. 4). Patients discharged using the Flex Hospitalist role had discharge orders entered an average of 213 minutes earlier and left the hospital an average of 201 minutes earlier than other inpatient HM discharges when measured after 1 year of the Flex Hospitalist role’s existence. In the second full year of the Flex Hospitalist role, 10.9% of the service’s discharges were completed by the Flex Hospitalist and maintained an average order time of 189 minutes earlier and an average discharge time of 167 minutes earlier than regular discharges.

**Fig. 5.**
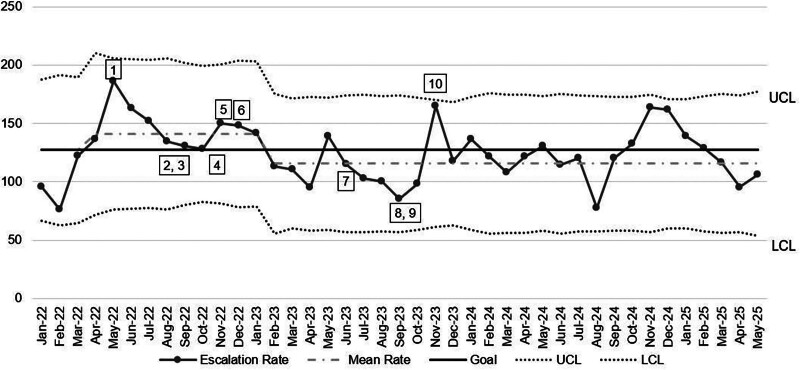
EOC rates to intermediate care unit or intensive care unit per 10,000 non–intensive care unit patient days. LCL, lower control limit; UCL, upper control limit. Intervention key: 1, single sign-on for EVS; 2, electronic nursing handoff; 3, 90-minute timer on ED track board; 4, clinical unit assignments; 5, hospitalist acceptance of ED admissions; 6, Flex Hospitalist; 7, expansion hours of hospitalist acceptance of ED admissions; 8, real-time demand capacity twice-daily huddles; 9, discharge lounge opening; 10, clinical bed assignments.

With the implementation of changes during this study, the average EOC rate was initially higher (141.24 per 10,000 non–intensive care unit days) than the previous year’s average (127.24) at the start of the first PDSA cycles. One year after the initiation of the study, the average decreased to 106.77 (Fig. 5).

The improvement in the primary outcome measure has been sustained for more than 1 year with no increase in balancing measures.

## DISCUSSION

Although patient flow through the ED plays a role, this project demonstrated that assessing the throughput systemically and addressing constraints to move people, information, and resources efficiently can have a significant impact on patient flow throughout the system. This project aimed to increase the percentage of patients admitted from the ED to the inpatient Med-Surg units within 90 minutes of the admission decision to 40%, from a baseline of 18%. The introduction of a hospitalist in the OC, who accepts admissions and places admission orders, and the expansion of this service from 8 to 16 hours per day were 2 key interventions that contributed most to the sustained improvement in the primary outcome measure. Because this is not a patient-facing or revenue-generating role, physician leaders must demonstrate the value of such interventions to hospital leaders. By including hospital leadership in the Steering Committee, the value of this role in both increasing downstream revenue (eg, decreasing LWBS rates and reducing admission turnaround times) and enhancing patient safety was repeatedly emphasized. The ED and HM division directors, as standing members of the Steering Committee, also disseminated these data and their impact on revenue and patient safety at physician leadership meetings. Because compensation and revenue plans vary widely, physician leaders should understand their services’ plans and be prepared to demonstrate the value of their QI work to their hospital and institutions, even in nonrelative value unit forms.

In previous studies, improvements to patient flow were diminished because only ED bottlenecks were addressed.^1,14,15^ By using a multidisciplinary approach, key interventions targeting multiple bottlenecks enabled further improvement in patient flow. With sustained interventions, the percentage of patients admitted within 90 minutes has remained greater than the 40% target for more than a year after the last intervention reported in this study. The correlation between the decrease in the LWBS rate and the positive shift in the centerline of the percentage of patients admitted within 90 minutes in November–December 2022 suggests that with improved patient flow through the hospital system, more patients are likely to receive timely care in the ED, resulting in a lower LWBS rate. The improvement in EOC around 1 year into the study period is likely influenced by 2 factors: (1) a hospitalist accepting admissions from the ED, allowing for quicker assessment of patient suitability for a Med-Surg unit, and (2) clinically experienced OC nurses taking over unit assignments.

This study included only patients admitted to the Med-Surg units on an inpatient pediatric HM team. This patient population accounts for a large proportion of hospital admissions (60%) managed by a single clinical service. One limitation to expanding the initiatives is the difference in admission/discharge workflows between the HM service and other subspecialties and surgical teams.

Some PDSA cycles resulted in abandoning the interventions. One example is the discharge lounge, which was limited by available hours, concerns related to infectious diseases and behavioral health, and a lack of buy-in from families/nursing staff due to the lounge’s limited resources. ED-based interventions aimed at rapid medical evaluations and increasing the availability of ED beds for patient assessment were inconsistently implemented due to staffing limitations. During times of high census, the Flex Hospitalist was pulled into primary staffing of pediatric patients instead of covering discharge duties. Another limitation was communicating frequent process changes, especially with a large volume of new hospital staff.

This study demonstrated that with a multidisciplinary approach to hospital throughput, specifically the admission process between the hospitalists and ED, significant improvement in meaningful metrics can be achieved and sustained without negatively affecting patient safety. This initiative led to increased collaboration between hospital departments, improving throughput and enhancing patients’ journeys through our hospital. Additional areas for study at our institution include expansion to other services, improving rounding practices, and targeting patient flow in additional departments, such as intensive care units and perioperative areas. Future studies could analyze the financial benefits of improved patient throughput in the hospital to increase stakeholder value.

## CONCLUSIONS

A multidisciplinary approach to hospital throughput, incorporating initiatives from the ED, inpatient units, and ancillary services, can significantly improve patient flow without negatively impacting patient safety.

## ACKNOWLEDGMENTS

The authors wish to acknowledge all the departments at Arkansas Children’s Hospital that were key to initiation and sustained improvement of our key metrics, including the Arkansas Children’s Emergency Medicine Section, Arkansas Children’s Pediatric Hospital Medicine Section, and Arkansas Children’s Operations Center, along with the inpatient nursing units and ancillary services, as critical contributors to the success of this work. The authors would also like to acknowledge Rick Pippin and John Forbus, process engineers at Arkansas Children’s Hospital, for their assistance with data analysis.

## Supplementary Material


