# Supplementary material for: A Multidisciplinary Quality Improvement Initiative to Improve Hospital Throughput in a Pediatric Tertiary Care Center
Source: Pediatr Qual Saf. 2025 Dec 1;10(6):e857. doi: 10.1097/pq9.0000000000000857 (PMC13169245; doi:10.1097/pq9.0000000000000857)
Supplement: Supplementary file 1 [file pqs-10-e857-s001.pdf]

Supplemental Figure 1: Details about Real Time Demand/Capacity (RTDC) Meetings

Meeting Times	Rationale for Meeting Times	Involved Personnel	Examples of Offered Solutions
9:40AM	After small unit and leader huddles were done and prior to the hospital wide safety huddle	Administration on Call, Emergency Dept, Nursing Supervisor, Operations Center Nurse, Safety & Security, Facilities Manager, Chief Nursing Office, Information Services, Patient Experience, Patient Safety, Transport, Laboratory, Infection Prevention, Respiratory Therapy, Radiology, Environmental Services, Maintenance Director, Social Work, Pharmacy, Outpatient Administration, Nutrition Services, Case Managers, Compliance, Risk Management/Legal Affairs, and Chief Quality & Safety Officer	The solutions offered in these sessions were typically assessing bed capacity in the hospital and determining which areas of the hospital might need to adapt their day (i.e. pausing non-emergent OR cases, ED diverting patients, inpatient team expediting discharges etc.) to maintain adequate patient throughput
3PM	Before shift changes occurred with the aim to provide enough time to gather data from the day and provide solutions for potential overnight and next day problems		
